# Liver X receptor alpha ensures blood-brain barrier function by suppressing SNAI2

**DOI:** 10.1038/s41419-023-06316-8

**Published:** 2023-11-28

**Authors:** D. Vacondio, H. Nogueira Pinto, L. Coenen, I. A. Mulder, R. Fontijn, B. van het Hof, W. K. Fung, A. Jongejan, G. Kooij, N. Zelcer, A. J. Rozemuller, H. E. de Vries, N. M. de Wit

**Affiliations:** 1 Amsterdam UMC location Vrije Universiteit Amsterdam, Department of Molecular Cell Biology and Immunology, De Boelelaan 1108, Amsterdam, the Netherlands; 2 Amsterdam Neuroscience, Amsterdam, the Netherlands; 3 Biomedical Primate Research Centre, Department of Neurobiology and Aging, Rijswijk, the Netherlands; 4 Amsterdam UMC location University of Amsterdam, Department of Biomedical Engineering and Physics, Meibergdreef 9, Amsterdam, the Netherlands; 5 Amsterdam UMC location University of Amsterdam, Epidemiology and Data Science, Meibergdreef 9, Amsterdam, The Netherlands; 6 Amsterdam Public Health, Methodology, Amsterdam, The Netherlands; 7 Amsterdam Infection and Immunity, Inflammatory Diseases, Amsterdam, The Netherlands; 8 Amsterdam UMC location University of Amsterdam Department of Medical Biochemistry, Meibergdreef 9, Amsterdam, the Netherlands; 9 Amsterdam UMC location University of Amsterdam, Cardiovascular Sciences and Gastroenterology and Metabolism, Meibergdreef 9, Amsterdam, the Netherlands; 10 Amsterdam UMC location Vrije Universiteit Amsterdam, Department of Pathology, De Boelelaan 1117, Amsterdam, the Netherlands

**Keywords:** Alzheimer's disease, Blood-brain barrier

## Abstract

In Alzheimer’s disease (AD) more than 50% of the patients are affected by capillary cerebral amyloid-angiopathy (capCAA), which is characterized by localized hypoxia, neuro-inflammation and loss of blood-brain barrier (BBB) function. Moreover, AD patients with or without capCAA display increased vessel number, indicating a reactivation of the angiogenic program. The molecular mechanism(s) responsible for BBB dysfunction and angiogenesis in capCAA is still unclear, preventing a full understanding of disease pathophysiology. The Liver X receptor (LXR) family, consisting of LXRα and LXRβ, was reported to inhibit angiogenesis and particularly LXRα was shown to secure BBB stability, suggesting a major role in vascular function. In this study, we unravel the regulatory mechanism exerted by LXRα to preserve BBB integrity in human brain endothelial cells (BECs) and investigate its role during pathological conditions. We report that LXRα ensures BECs identity via constitutive inhibition of the transcription factor SNAI2. Accordingly, deletion of brain endothelial LXRα is associated with impaired DLL4-NOTCH signalling, a critical signalling pathway involved in vessel sprouting. A similar response was observed when BECs were exposed to hypoxia, with concomitant LXRα decrease and SNAI2 increase. In support of our cell-based observations, we report a general increase in vascular SNAI2 in the occipital cortex of AD patients with and without capCAA. Importantly, SNAI2 strongly associated with vascular amyloid-beta deposition and angiopoietin-like 4, a marker for hypoxia. In hypoxic capCAA vessels, the expression of LXRα may decrease leading to an increased expression of SNAI2, and consequently BECs de-differentiation and sprouting. Our findings indicate that LXRα is essential for BECs identity, thereby securing BBB stability and preventing aberrant angiogenesis. These results uncover a novel molecular pathway essential for BBB identity and vascular homeostasis providing new insights on the vascular pathology affecting AD patients.

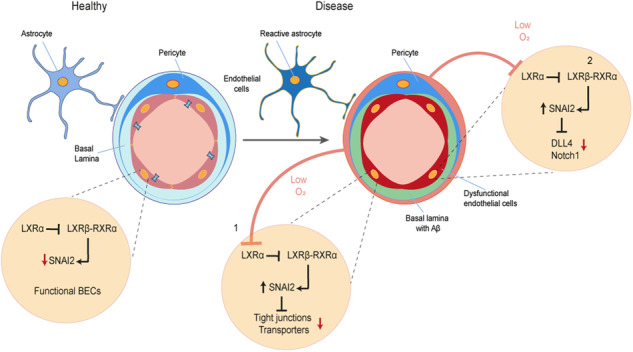

## Introduction

The blood-brain barrier (BBB) is an important cellular interface that is essential for the maintenance of brain homeostasis. The BBB is composed of specialized brain endothelial cells (BEC), which form a physical barrier between the blood and the brain. The highly differentiated BECs form a continuous barrier by means of tight junctions (TJs), consisting of proteins such as claudin-5 (CLDN5) and occludin (OCLDN), which limit the exchange of molecules and cells [1]. In addition, BECs express specialized influx transporters, including the glucose transporter and efflux transporters such as the ATP binding cassette (ABC) transporters. Together they tightly regulate the bidirectional transcellular transport of metabolites, ensuring the brain metabolic demand is met [2]. By forming this tight and selective barrier, the BBB protects the central nervous system (CNS) from unwanted neurotoxic compounds or cells.

Under healthy conditions, the specialized and highly differentiated BECs identity is secured by multiple signalling pathways including the Wnt/β-catenin and many others [3, 4]. However, during disease, BECs display remarkable phenotypic plasticity, highlighted by their ability to undergo endothelial-to-mesenchymal transition (EndMT) [5, 6]. During EndMT, transcription factors such as SNAI1 and SNAI2 drive endothelial cells to lose their specific markers (e.g. CLDN5, OCLDN) and progressively acquire a mesenchymal phenotype [7]. This associates with elongated BEC morphology, loss of cell-cell junctions and polarity as well as gaining motility, invasive and contractile properties [8, 9]. Although EndMT is essential during embryonic development, this process has also been associated with different CNS disorders, including multiple sclerosis and cerebral cavernous malformation, thereby contributing to BBB dysfunction [7, 10].

BBB dysfunction is increasingly recognized as a critical factor in Alzheimer’s disease pathophysiology [11–16]. More than half of the AD patients suffer from capillary cerebral amyloid angiopathy (capCAA), which is associated with tight junction and ABC transporters reduction (e.g. ABCG2), thereby exacerbating BBB dysfunction and aggravating AD pathology [17–21]. CapCAA is characterized by the perivascular accumulation of amyloid beta (Aβ) in cortical capillaries, although it can affect larger vessels as well [22, 23]. Moreover, astrocytes surrounding the affected capillaries express higher levels of Angiopoietin-like 4 (ANGPTL4), a pro-angiogenic factor induced by hypoxia [18–20]. Interestingly, hypoxia is an important driver of EndMT in various endothelial cells via the induction of SNAI1 and SNAI2 [24–28]. However, despite extensive investigation of the mechanisms underlying hypoxia-related EndMT in non-brain derived endothelial cells, the detailed molecular and regulatory alterations in BECs have not been fully elucidated.

Recently, the family of Liver x receptors (LXRs) has been implicated in the process of epithelial-to-mesenchymal transition [29]. LXRs are members of the nuclear receptor family of ligand-activated transcription factors, and consist of two isoforms, LXRα and LXRβ. They regulate the expression of several genes involved in cholesterol and fatty acid metabolism. Once activated, LXRs binds as a heterodimer with the obligate partner retinoid x receptor (RXR) to LXR-responsive elements and promote gene expression [30, 31]. Important downstream target genes of the LXR pathway are ABC transporter genes (ABCA1 and ABCG1), Apolipoprotein E, and the E3 ubiquitin ligase IDOL [32]. Importantly, the LXR-RXR heterodimers are considered permissive, and thus may be activated by agonists of either heterodimeric receptor [33, 34]. Apart from cholesterol metabolism, LXRs are important for other biological functions, including inflammation and BBB function [35–40].

We previously reported that LXRα is important for maintaining BBB integrity and its immune quiescence under normal and inflammatory conditions [41]. In the present study, we aim to unravel the regulatory mechanism exerted by LXRα to preserve BBB integrity. Here, we report that LXRα secures BEC identity. Our data suggest that LXRα limits the interaction of LXRβ with RXR, thereby inhibiting the expression of SNAI2. The knockdown of *LXRα* in BECs results in increased *SNAI2* expression, loss of BEC markers, and aberrant angiogenesis via the suppression of the DLL4-NOTCH signalling pathway. Moreover, we show that hypoxia specifically affects LXRα and recapitulates the effects observed in LXRα knockdown cells. Finally, we show an increased expression of *SNAI2* in Aβ-affected vessels in post-mortem tissue from AD patients with and without capCAA. Collectively, our findings elucidate a novel mechanism via which LXRα secures BBB integrity and how its impairment might contribute to the observed BBB dysfunction in AD patients with capCAA.

## Materials and methods

### Cell culture

#### Human cerebral microvascular endothelial cell line hCMEC/D3

The human cerebral microvascular endothelial cell line hCMEC/D3 was kindly provided by Dr. Couraud [42] (Institute Cochin, Université Paris Descartes, Paris France). hCMEC/D3 cells were grown in Endothelial Cell Growth Basal Medium-2 (EBM-2), including supplement components according to the manufacturer’s instructions (EGM-2) (Lonza, Basel, Switzerland). All hCMEC/D3 cell culture plates were coated with type I collagen (Invitrogen, Thermo Fisher Scientific, Leusden, The Netherlands). Cultures were grown to confluence at 37 °C in 5% CO_2_. hCMEC/D3 cells were detached with trypsin/EDTA in PBS (Gibco, Thermo Fisher Scientific, Leusden, The Netherlands). At the start of the hypoxia experiment, hCMEC/D3s were sterol-starved for 12 h in RPMI-1640 medium (Gibco, Thermo Fisher Scientific, Leusden, The Netherlands) supplemented with Lipoprotein Deficient Serum from human plasma (Sigma-Aldrich, Diegem, Belgium) and cultured for 48 h at 37 °C in 1% O_2_. Cells were regularly tested for mycoplasma infection.

#### Neural crest-derived brain pericytes (iBPC)

Human induced pluripotent stem cells (hiPSC) were differentiated to neural crest (NC)-derived brain pericytes (iBPC) using previously published protocols [43, 44]. Briefly, Gibco episomal hiPSC line (Gibco, #A13700, Thermo Fisher Scientific, Leusden, The Netherlands) was cultured in mTeSR Plus medium (STEMCELL Technologies, Vancouver, Canada) on vitronectin-coated plates (Invitrogen, Thermo Fisher Scientific, Leusden, The Netherlands). To start the differentiation, hiPSCs were passaged as single cells and seeded at a density of 2 ×10^5^ cells/cm2 onto Matrigel-coated plates and cultured in NC induction medium, consisting of DMEM/F12 GlutaMAX™ (Gibco, Thermo Fisher Scientific, Leusden, The Netherlands), 1× B27 (Gibco, Thermo Fisher Scientific, Leusden, The Netherlands), 0.5% BSA and 3 µM CHIR 99021 (Tocris, Bristol, United Kingdom) for 5 days. The resulting NC cells were passaged as single cells and seeded at a density of 2.5 ×10^4^ cells/cm2 onto 0.1% gelatin-coated plates and cultured in pericyte medium (ScienCell, Carlsbad, CA, USA) for 5 days for BPC specification. Immunocytochemistry and mRNA evaluation of PDGFRβ, NG2, CD13, FOXF2, FOXC1, CD146, vitronectin confirmed their pericyte identity. iBPC were used for sprouting experiments between passages 2 and 4.

### Lentiviral short hairpin RNA for LXRα, LXRβ and HIF-1α knockdown

Selective gene knockdown was obtained by using a vector-based short hairpin RNA (shRNA) technique as previously described [45]. Recombinant lentiviruses were produced by co-transfecting sub-confluent HEK293T cells with the specific expression plasmids and packaging plasmids (pMDLg/pRRE, pRSV-Rev and pMD2G) using calcium phosphate as a transfection reagent. HEK293T cells were cultured in Dulbecco’s modified eagle medium (DMEM) supplemented with 10% fetal calf serum (FCS) and 1% penicillin/streptomycin. Cells were cultured at 37 °C in 5% CO_2_. Infectious lentiviral particles were collected 48 hours after transfection and stored at −80 °C upon further use. The knockdown (KD) efficiency of all 5 constructs for each gene was tested, and the most effective constructs were used in subsequent experiments. For LXRα (NR1H3), TRC22237 was selected, with encoding sequence 5’-GTGCAGGAGATAGTTGACTTT-3’ that targets nucleotides 1043–1063 of the NM_005693.3 RefSeq. For *LXR*β (NR1H2) the most effective construct was TRC275326, encoding the sequence 5’-GAAGGCATCCACTATCGAGAT-3’ that targets nucleotides 1193–1213 of the NM_007121.5 RefSeq. For *HIF-1A* (HIF1A) the most effective construct was TRCN0000010819, encoding the sequence 5’-TGCTCTTTGTGGTTGGATCTA-3’. Subsequently, lentiviruses expressing LXRα, *LXRβ* or *HIF-1α* specific shRNA were used to transduce hCMEC/D3 cells. Control cells were generated by transduction with lentivirus expressing nontargeting shRNA (SHC002, Sigma-Aldrich, St Louis, MO). Twenty-four hours after infection of hCMEC/D3 cells with the shRNA-expressing lentiviruses, stable cell lines were selected by puromycin treatment (2 μg/ml, Sigma-Aldrich, Diegem, Belgium). The knockdown efficiency was determined by quantitative real-time PCR (qRT-PCR) and Western blot. The primer sequence is listed in Supplementary Table 1. At the start of the experiment, transduced hCMEC/D3s were treated with the LXRs agonist GW3965 (1 μM, Sigma-Aldrich, Diegem, Belgium), LXRs antagonist GSK2033 (1 µM, R&D systems, Minneapolis, MN, USA), retinoic acid (5 µM, Sigma-Aldrich, Diegem, Belgium), *RXRα* antagonist PA452 (1 µM, MCE, New Jersey, USA) or dimethyl sulfoxide (DMSO) as vehicle control for 48 or 72 h in EGM-2 media (Lonza, Basel, Switzerland).

### RNA isolation and qRT-PCR

Recombinant hCMEC/D3 cells (1×10^6^ cells/ml) transduced with either LXRα shRNA, LXRβ shRNA, or non-targeting shRNA were seeded in 24-well plates in culture medium. RNA was isolated using the TRIzol® method (Life Technologies, Bleiswijk, The Netherlands) and cDNA was synthesized using the Reverse Transcription System kit (Promega, Leiden, The Netherlands). Sequences of primers used are listed in Supplementary Table 1. Quantitative Reverse Transcriptase PCR (qRT-PCR) was carried out using SYBR green master mix (Applied Biosystems, Waltham, MA, USA) and a Step One Plus detection system (Applied Biosystems). Quantification of gene expression was accomplished using the comparative cycle threshold method. Expression levels were normalized to Glyceraldehyde 3-phosphate dehydrogenase (GAPDH) or Ribosomal Protein Lateral Stalk Subunit P0 (RPLP0) expression.

### RNA sequencing-based transcriptional profiling and analysis

The hCMEC/D3 cells were cultured and treated as described above. Total RNA was extracted using TRIzol reagent (Invitrogen, Carlsbad, CA, USA) and converted into strand-specific cDNA libraries using the TruSeq Stranded mRNA sample preparation kit (Illumina, San Diego, CA, USA) according to the manufacturer’s instructions. Briefly, polyadenylated RNA was enriched using oligo-dT beads and subsequently fragmented, random primed and reverse transcribed using SuperScript II Reverse Transcriptase (Invitrogen, Carlsbad, CA, USA). Second-strand synthesis was performed using Polymerase I and RNaseH with the replacement of dTTP for dUTP. The generated cDNA fragments were 3’ end adenylated, ligated to Illumina paired-end sequencing adapters, and subsequently amplified by 12 cycles of PCR. The libraries were analysed on a 2100 Bioanalyzer using a 7500 chip (Agilent, Santa Clara, CA, USA) and subsequently sequenced with 65 base single reads on a HiSeq2500 using V4 chemistry (Illumina, San Diego, CA, USA) Transcripts were aligned to the Human Feb. 2009 (GRCh37/hg19) assembly using TopHat (version 2.1) [46]. Gene expression sets were prepared using ICount, which is based on HTSeq-count. Uniquely mapped reads were normalized to 10 million reads followed by log2 transformation. In order to avoid negative normalized values, 1 was added to each gene expression value. Data were analysed using Gene Set Enrichment Analysis software [47, 48] (University of California San Diego, San Diego, CA, USA) and the differentially expressed gene sets (nominal *p* value < 0.05) displayed. The results of the gene set enrichment analysis are displayed in Supplementary Table 2. The heat map was created using Heat mapper online tool, plotting the count per million per sample [49]. The RNA-seq data were analysed as follow, transcripts with more than 2 counts in 3 or more of the samples were kept, for data normalization TMM (edgeR), weighted trimmed mean of M-values (to the reference) was used [50], the data were annotated using biomaRt, using Ensembl. The count data was transformed to log2-counts per million (logCPM) using voom, estimating the mean-variance relationship and the differential expression was assessed using a moderated t-test using the linear model framework from the limma package and the adjusted *p* value calculated by using FDR, Benjamini-Hocheberg correction (Supplementary Table 3). Differentially expressed genes (FDR adj. *p* value < 0.05 and Log2 fold change of 0.5) were displayed in a volcano plot.

### Western blot and nuclear fractionation

After washing with ice-cold phosphate-buffered saline (PBS), hCMEC/D3 cells were lysed with cell lysis buffer (Cell Signaling Technology, Boston, MA, USA) containing a protease and phosphatase inhibitor cocktail (Roche, Almere, The Netherlands, and Cell Signaling Technology, Boston, MA, USA, respectively) on ice, following the manufacturer’s instructions. Nuclear fractions were isolated using the NE-PER extraction kit (Thermo Fisher Scientific, Rockford, IL, USA), following the manufacturer’s guidelines. All samples were diluted in sample buffer (BioRad Hercules, CA, USA) (65.8 mM Tris-HCl, pH 6.8, 2.1% SDS, 26.3% (w/v) glycerol, 0.01% bromophenol blue) and heated to 95 °C for 3 min. For whole cell lysates, hCMEC/D3 were removed from the media and lysed in sample buffer. Lysates were separated on SDS-PAGE followed by transfer to nitrocellulose for immune-blot analysis. Blots were blocked for 1 h at room temperature with blocking buffer (Azure Biosystems, Inc, Sierra CT, Dublin, CA, USA). Subsequently, membranes were incubated in blocking buffer containing 0.1% Tween-20 with antibodies against LXRα (R&D Systems, Minneapolis, MN, USA), Laminin (MP Biomedicals, Sant Ana, CA, USA) and GAPDH (Proteintech, Manchester, United Kingdom). Primary antibodies were detected and quantified by incubation with IRDye secondary antibodies (LI-COR) and use of Azure Sapphire Biomolecular Imager (Azure Biosystems, Inc, Sierra CT, Dublin, CA, USA).

### Immunofluorescence microscopy

hCMEC/D3 cells were seeded in 8 well µ-slides (Ibidi, München, Germany) and treated as described in the cell culture section. Cells were fixed with 4%, 1.6% paraformaldehyde, or ice-cold methanol (Sigma-Aldrich, Saint Louis, MO, USA) and then permeabilized for 5 min using 0.05% Triton-X100 in PBS (Sigma-Aldrich, Saint Louis, MO, USA). Unspecific binding was prevented with 5% normal goat serum. Cells were then incubated with mouse anti-claudin-5 (Santa Cruz, Dallas, TX, USA), mouse anti-SNAI2 (Abcam, Cambridge, United Kingdom), mouse anti-CD31 (DAKO, Naestved, Denmark), Delta-4 Antibody (G-12) (Santa Cruz, Dallas, TX, USA) and rabbit anti-Zonulin-1 (*ZO1*) (Thermo Fisher Scientific, Rockford, IL, USA). Primary antibodies were visualized using goat anti-mouse Alexa 555/488 (Molecular Probes, Eugene, OR, USA). Nuclei were visualized using Hoechst (Molecular Probes, Eugene, OR, USA). Stainings were imaged using the Leica SP8 microscope (Leica, Mannheim, Germany) or LIPSI Ti2 (Nikon, Tokyo, Japan)

### 3D in vitro sprouting assay

Spheroids were generated for the sprouting assay. In brief, hCMEC/D3 and hiPSCs pericytes were resuspended in a ratio of 20:1 in EGM-2 medium containing 0.25% methylcellulose (4.000 cP, Sigma-Aldrich, Saint Louis, MO, USA). To form spheroids, the mixture of cells was seeded in a 24 well plate and flipped upside down. After 24 h, the spheroids were collected and resuspended in 1,5 mg/ml collagen Type I rat tail mixture (Enzo science, Farmingdale, NY, USA) and plated in a 24-well plate upside down until complete polymerization. After 30 min, EGM-2 medium was administered and wells were incubated at 37 °C and 20% O_2_, 5% CO_2_ for 5 days or at 1% O_2_, 5% CO_2_ for 5 days. Images were taken using the Nikon LIPSI Ti2 confocal spinning disk imaging system (Nikon, Tokyo, Japan), 10 x objective, and adjusted for brightness/contrast in ImageJ. Sprouting number and length were analysed using the ImageJ plugin NeuronJ .

### Immunohistochemistry on postmortem human brain tissue

Brain tissue from 5 patients with clinically diagnosed and neuropathologically confirmed capCAA, 6 AD and 6 non-demented control (NDC) cases without neurological diseases was obtained after autopsy (post-mortem delay < 8 h) and immediately frozen in liquid nitrogen (in collaboration with the Netherlands Brain Bank, Amsterdam). The Netherlands Brain Bank received permission from the ethical committee of the VU University Medical Center Amsterdam, the Netherlands to perform autopsies, for the use of the material and for access to medical records for research purposes. Cortical grey matter samples from the superior occipital gyrus (SOG) were selected and used for staining. All patients and controls, or there next of kin, had given informed consent for autopsy and use of their brain tissue for research purposes. Clinical data are presented in Supplementary Table 4.

For immunohistochemical analysis, 5 µm thick cryosections of frozen brain tissues were fixed in ice-cold acetone for 10 min. After washing with PBS, sections were incubated overnight at 4 °C with primary antibodies against SNAI2 (Abcam, Cambridge, United Kingdom). Subsequently, sections were washed with PBS and incubated with Envision Dual Link (DAKO, Glostrup, Denmark) for 1 h at room temperature, followed by visualization with the peroxidase substrate 3,3′-diaminobenzidine (DAKO, Glostrup, Denmark). Sections were incubated with hematoxylin (Sigma-Aldrich, Saint Louis, MO, USA) for 1 min and thoroughly washed with tap water for 10 min. Ultimately, sections were dehydrated with ethanol followed by xylene (Sigma-Aldrich, Saint Louis, MO, USA) and mounted with Entellan (Merck, Darmstadt, Germany). Immunofluorescent labelling was performed as follows: after fixation in ice-cold acetone for 10 min, the sections were incubated for 30 min with 10% normal goat serum and 0.1% Triton X-100 (Sigma-Aldrich, Saint Louis, MO, USA) and afterwards incubated overnight at 4 °C with antibodies against SNAI2 (Abcam, Cambridge, United Kingdom) or ANGPTL4 (Abcam, Cambridge, UK), and sections were stained with UEA-1 (Vector Lab, Burlingame, CA, USA). The primary antibodies were visualized by incubation with goat anti-mouse Alexa 555 (Molecular Probes, Eugene, OR, USA), donkey antirabbit Alexa 647 (Molecular Probes, Eugene, OR, USA) or Streptavidin 488 (Molecular Probes, Eugene, OR, USA) for 1 h at RT. Next, to visualize Aβ aggregates, sections were incubated for 5 min with Thioflavin-S (Sigma-Aldrich, Saint Louis, MO, USA) and washed with ethanol afterwards. After washing with PBS, Hoechst (Molecular Probes, Eugene, OR, USA) was used for nuclear staining and slides were mounted in Mowiol (Sigma-Aldrich, Saint Louis, MO, USA).

### Image acquisition and analysis

Images of the DAB-stained tissue were obtained using a DM6000 (Leica, Mannheim, Germany), 4 random regions of interest (ROIs) were collected per sample and the results presented as average staining intensity per section. Fluorescent images were obtained using an Olympus VS200 (Olympus, Tokyo, Japan) slide scanner or a SP8 confocal microscope (Leica, Mannheim, Germany). Five specific ROIs with a Z-stacks of 6 µm and a 60x magnification were recorded and the results are presented as average staining intensity per section. Image deconvolution and analysis were done using Huygens Professional 21.10 software (Scientific Volume Imaging B.V., Hilversum, The Netherlands) and NIS elements (version 5.30.03, Nikon Europe B.V., Amsterdam, The Netherlands) or ImageJ (U.S. National Institutes of Health, Bethesda, MD, USA).

### Statistical analysis

Data were statistically analysed using GraphPad Prism v9 (GraphPad Software, La Jolla, CA, USA). Ordinary one-way ANOVA (3 groups), or two-tailed paired or unpaired student t-test (2 groups) with original FDR method of Benjamini and Hochberg multiple comparison correction were used for normally distributed data sets. The Kruskal-Wallis (3 groups) or paired Wilcoxon signed-rank test and Mann-Whitney (2 groups) analysis with original FDR method of Benjamini and Hochberg correction was used for non-parametric data sets. *P < 0.05, **P < 0.01, ***P < 0.001 (adjusted *p* value).

## Results

### LXRα deficient BECs display a profound loss of brain endothelial markers

**Fig. 1:**
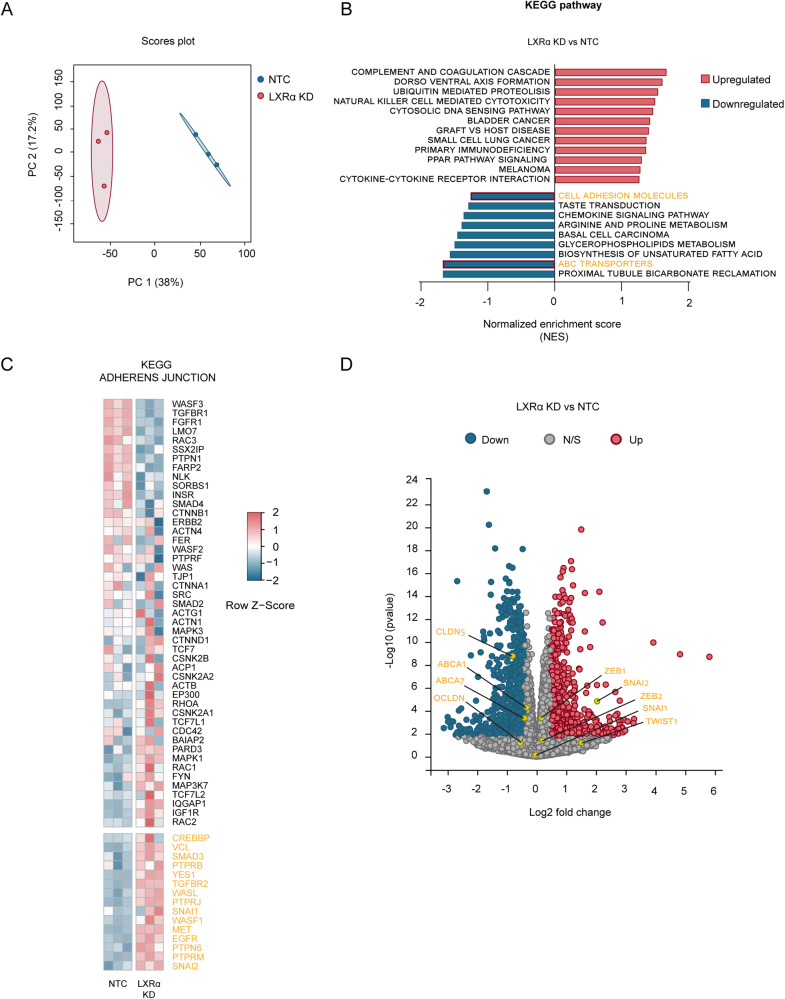
LXRα deficient BECs display a profound loss of brain endothelial markers. **A** Principal component analysis (PCA) showing different clustering of hCMEC/D3 cells transduced with shLXRα (LXRα KD) or shNTC (NTC). **B** Gene set enrichment analysis of LXRα KD vs NTC cells, presenting significantly different gene sets (nominal *p* value < 0.01). **C** Heat-map of KEGG adherens junctions, in yellow the genes with a core enrichment. **D** Volcano plot showing differentially expressed genes of LXRα KD vs NTC cells. In yellow BEC markers and transcription factors of interest. The data highlighted (Red and Blue) represent all the significantly differentially expressed genes (adjusted *p* value < 0.05) with a Log2 Fold change of at least 0.4 (which correspond to at least −30% difference) to identify significantly enriched biological processes.

To investigate the underlying mechanism of BBB dysfunction caused by the depletion of *LXRα* in BECs, we compared the transcriptional profile of control (shRNA non-targeting (NTC)) and LXRα knockdown cells (LXRα KD) using RNA-seq. The transcriptional profile of the LXRα KD cells showed remarkable differences with NTC cells as highlighted in the principal component analysis (Fig. 1A). An in-depth analysis revealed a total of 4223 differentially expressed genes between the two groups, of which 2038 were up- and 2185 down-regulated in LXRα KD cells compared to NTC cells. The Gene Set Enrichment Analysis (GSEA) showed 12 significantly upregulated gene set (*p* < 0.01) and 9 significantly downregulated gene set (*p* < 0.01) (Fig. 1B). Among the downregulated gene set, we recognized the KEGG-ABC transporters and KEGG-cell adhesion molecules (Fig. 1B). These gene sets include important brain endothelial markers such as ABCA1, ABCG1, OCLDN and CLDN5 (Supplementary Fig. 1).

Since none of the significantly different gene sets could explain the loss of BEC markers, we explored the gene sets according to their Normalized Enrichment Score. The KEGG-adherens junction, ranked as one of the most different GS between NTC and LXRα KD cells. Among the genes present in this set, we recognized the transcription factors SNAI1 and SNAI2, which were increased in LXRα KD cells (Fig. 1C). The volcano plot shows a 4-fold increase of *SNAI2 (p* < 0.05*)* but no statistical difference in *SNAI1* expression (Fig. 1D). Collectively, these results indicate that silencing of LXRα reduces the expression of BEC markers and that this mechanism is potentially driven by SNAI2.

### LXRα constitutively inhibits SNAI2 expression in brain endothelial cells

**Fig. 2:**
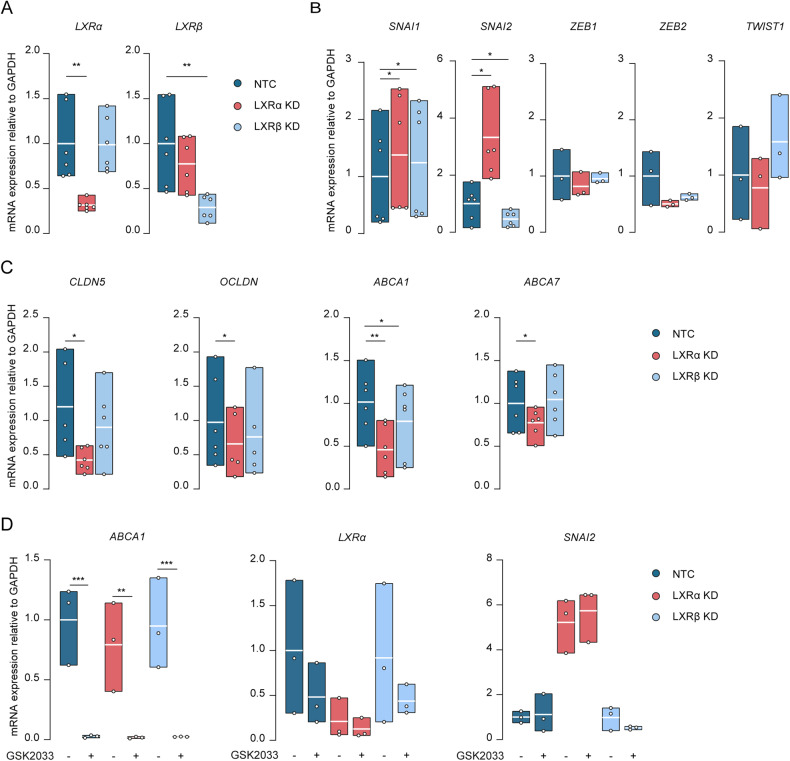
LXRα constitutively inhibits SNAI2 expression in brain endothelial cells. **A** Validation of knockdown of *LXRα* and *LXRβ* in LXRα/β KD cells at mRNA level. **B** mRNA levels of transcription factors measured in LXRα/β KD cells by qRT-PCR. **C** mRNA levels of BEC markers measured in LXRα/β KD cells by qRT-PCR. **D** *ABCA1*, *LXRα*, *SNAI2* mRNA levels measured by qRT-PCR in LXRα/β KD cells treated with GSK2033 (1 µM for 72 h). Values were normalized using GAPDH and plotted as fold change of NTC. Data presented are the mean of triplicate values of minimum three independent experiments. Statistical analysis was performed using paired Student’s t-test or ordinary one-way ANOVA with original FDR method of Benjamini and Hochberg correction where *p < 0.05, **p < 0.01, ***p < 0.001.

We next set out to validate the increase of SNAI genes observed in the RNA-seq of LXRα KD cells, via targeted quantitative mRNA analysis. Furthermore, we generated specific LXRβ KD BECs to elucidate whether the regulation of *SNAI1* and *SNAI2* expression is LXR isoform specific. The interference with shRNA significantly and specifically decreased the mRNA expression of *LXRα* (*p* = 0.008) and *LXRβ* (*p* = 0.005) in BECs (Fig. 2A). The protein content of LXRα was also decreased compared to NTC BECs (Supplementary Fig. 1). LXRα silencing increased the expression of *SNAI2* 3-fold (*p* = 0.015) and *SNAI1* to a lesser extent (*p* = 0.019) (Fig. 2B). This change in SNAI2 was LXRα specific as silencing of LXRβ failed to increase *SNAI2* expression (Fig. 2B). No significant differences were found regarding the levels of other transcription factors involved in EndMT (e.g., *ZEB1*, *ZEB2*, *TWIST1*) (Fig. 2B). We next validated the expression of BEC markers. LXRα KD cells resulted in a significant downregulation of the BEC-specific markers *CLDN5* (*p* = 0.044), *OCLDN* (*p* = 0.047), *ABCA1* (*p* = 0.002) and *ABCA7* (*p* = 0.049) (Fig. 2C). These markers were unchanged in LXRβ KD cells, with the exception of *ABCA1* (*p* = 0.049) (Fig. 2C).

To decipher whether the regulatory action exerted by LXRα on *SNAI2* expression is related to its activity, we treated NTC, LXRα and LXRβ KD cells with a broad LXR antagonist GSK2033 (1 µM for 72 h). Treatment of BECs with GSK2033 resulted in complete inhibition in the transcription of the LXR target gene *ABCA1* in all three conditions (NTC (*p* = 0.001), LXRα KD (*p* = 0.003), LXRB KD (p = 0.001)) (Fig. 2D). Moreover, inhibiting the activity of LXRα KD cells (by treating the LXRβ KD cells) did not result in an increase of *SNAI2*. Together, these results suggest that LXRα is essential to inhibit SNAI2 but that this likely involves a ligand-independent and indirect mechanism.

### LXRα prevents LXRβ-RXR interaction in brain endothelial cells

**Fig. 3:**
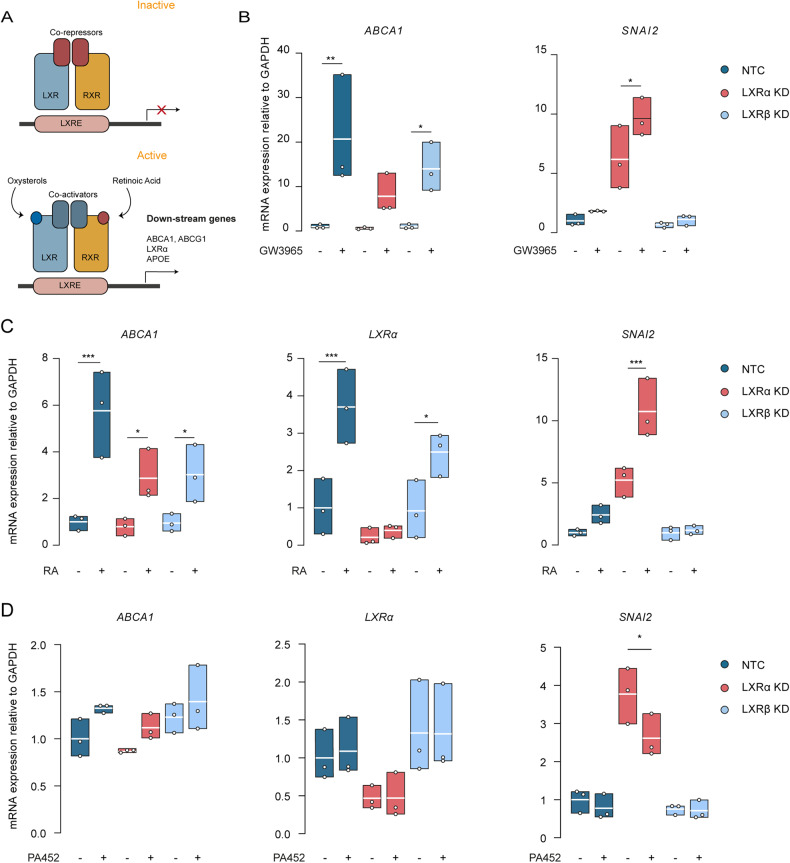
LXRα prevents LXRβ-RXR interaction in brain endothelial cells. **A** Schematic overview of the LXR pathway activation, which highlights the interaction between LXRs and RXR. **B** *ABCA1*, *SNAI2* mRNA levels measured by qRT-PCR in LXRα/β KD cells treated with GW3965 (1 µM for 48 h). **C** ABCA1, *LXRα, SNAI2* mRNA levels measured by qRT-PCR in LXRα/β KD cells treated with retinoic acid (5 µM for 72 h). **D** *ABCA1*, *LXRα, SNAI2* mRNA levels measured by qRT-PCR in LXRα/β KD cells treated with PA542 (1 µM for 48 h). Values were normalized using GAPDH and plotted as fold change of NTC. Data presented are the mean of triplicate values of three independent experiments. Statistical analysis was performed using ordinary one-way ANOVA with the original FDR method of Benjamini and Hochberg correction where *p < 0.05, **p < 0.01, ***p < 0.001.

As permissive receptors, both LXRs and RXRs are activated by oxysterols and retinoic acid (RA) [51], thereby inducing gene expression of both pathways (Fig. 3A). To further dissect the regulatory circuit underlying *LXRα* and *SNAI2* expression, we first treated BECs with the pan LXR agonist GW3965 (1 µM for 48 h) and investigated the expression of LXR target genes. GW3965 significantly increased the expression of *ABCA1* in NTC (*p* = 0.005) and LXRβ KD cells (*p* = 0.030) but not in LXRα KD cells (Fig. 3B). In parallel, the relative *SNAI2* expression, which was already substantially higher in LXRα KD cells, was significantly further increased upon stimulation (*p* = 0.020) (Fig. 3B).

To investigate whether activation of RXR induces LXR target gene expression, we next treated the cells with retinoic acid, which is an agonist of the RXR pathway (5 µM for 72 h). RA was able to activate the LXR pathway in NTC as shown by the significant increase of *ABCA1* (*p* < 0.001) and *LXRα* (*p* < 0.001). More importantly, the treatment with RA significantly increased the expression of *SNAI2* in LXRα KD cells (*p* < 0.001), while the compound had no effect on *SNAI2* expression in NTC and LXRβ KD cells (Fig. 3C).

To test whether the interaction of LXRβ and RXR is responsible for the increased *SNAI2* expression observed in LXRα KD cells, we treated BECs with a specific inhibitor for RXRα (PA452, 1 µM for 48 h) [52]. PA452 significantly decreased *SNAI2* expression (*p* = 0.020) in LXRα KD cells, while it did not affect the expression in NTC and LXRβ KD cells (Fig. 3D). Furthermore, the RXRα inhibitor had no effect on *ABCA1* and *LXRα* expression in neither of the BECs. Collectively these findings suggest a model in which the presence of LXRα prevents LXRβ-RXR interaction thereby counteracting *SNAI2* expression.

### LXRα regulates DLL4-Notch signalling

**Fig. 4:**
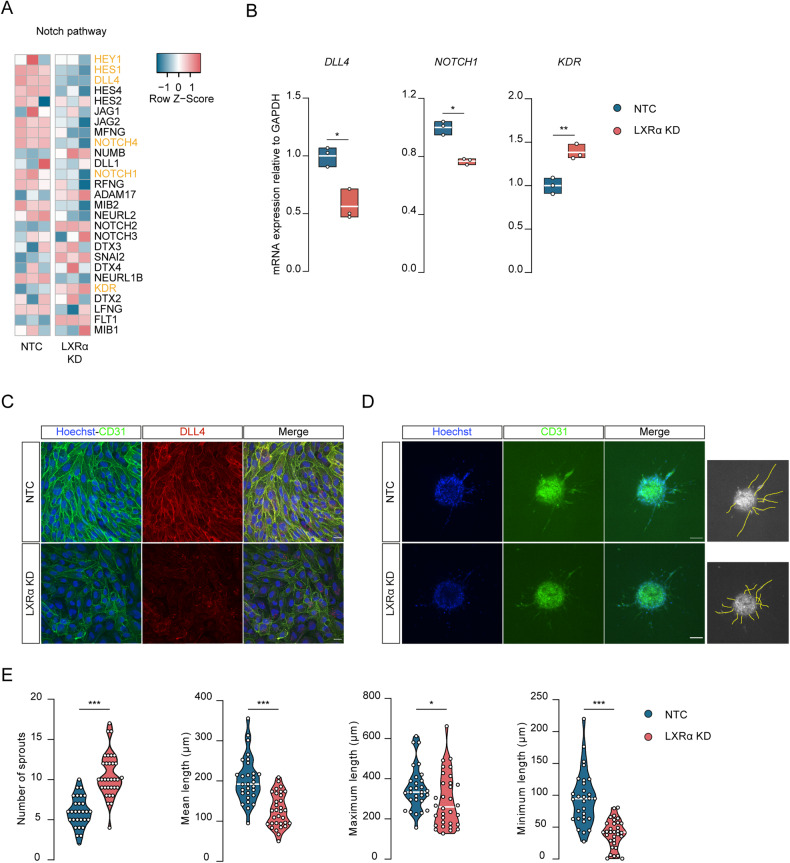
LXRα regulates DLL4-Notch signaling. **A** Heat-map of the Notch pathway components. In yellow the genes involved in angiogenesis. **B** mRNA levels of *DLL4*, *NOTCH1* and *KDR* measured by qRT-qPCR in LXRα KD cells, normalized to GAPDH, and plotted as fold change compared to NTC. **C** Immunostaining of sprouting spheroids. Hoechst (blue) CD31 (green) and DLL4 (red). Scale bar 20 µm. Data presented are the mean of triplicate values SEM of three independent experiments. **D** Representative images of sprouting assay of NTC (*N* = 28) and LXRα KD (*N* = 31) with relative analysis (yellow tracing). Hoechst (blue) and CD31 (green). Scale bar 100 µm. **E** Quantification of total number of sprouts and mean length, maximum and minimum length expressed per condition. Statistical analysis was performed using paired or unpaired Student’s t-test with Welch’s correction where *p < 0.05, **p < 0.01, ***p < 0.001.

The transcription factor *SNAI2* is directly responsible for the loss of endothelial markers (e.g. CLDN5, CD31) and aberrant angiogenesis via the suppression of DLL4-NOTCH signalling in human umbilical cord endothelial cells [53]. In light of this, we next assessed the NOTCH pathway in LXRα KD cells. Our RNA-seq data indicated a significant (*p* < 0.05) downregulation of key players in the Notch pathway (e.g. *DLL4*, *NOTCH1*, *NOTCH4, HES1*, *HEY1*) as depicted in the heat map (Fig. 4A). We confirmed these findings in LXRα KD cells, where we determined a significant decrease of both *DLL4* (*p* = 0.018) and *NOTCH1* transcripts (*p* = 0.019) in comparison to control cells (Fig.4B). *KDR* in contrast, which is the gene encoding for the vascular endothelial grow factor receptor 2 (*VEGFR2*) and essential during the tip-cell formation process, was increased in LXRα KD cells (*p* = 0.002). No significant differences were found for the other NOTCH ligands measured such as JAG1 and JAG2 (Supplementary Fig. 1). Moreover, DLL4 and NOTCH1 expression did not change upon GW3965 stimulation (Supplementary Fig. 1). We further confirmed the decrease of DLL4 and CD31 protein content in LXRα KD cells, which was accompanied by a corresponding loss of cellular polarization (Fig. 4C). To assess whether the increase of *SNAI2* expression in LXRα KD cells modulates Notch signalling, we performed a sprouting assay (Fig. 4D). LXRα KD cells showed a significant increase in the number of sprouts (*p* < 0.001), while these newly formed sprouts were on average shorter than those in NTC cells (*p* < 0.001) (Fig. 4E). All together, these data highlight the importance of LXRα in the DLL4-NOTCH axis maintenance and associated vessel formation.

### Hypoxia selectively suppresses LXRα in BECs

**Fig. 5:**
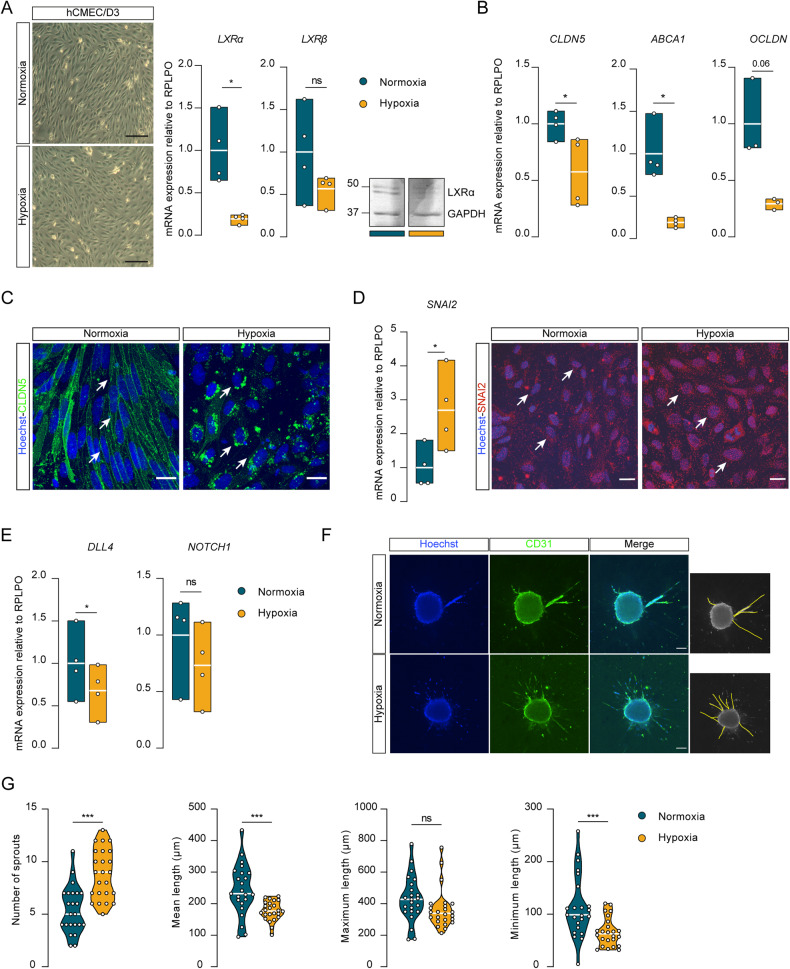
Hypoxia selectively suppresses LXRα in BECs. **A** Representative image of hCMEC/D3 cells after 48 h in 1% O_2_. Scale bar 200 µM. The mRNA levels of *LXRα* and *LXRβ* measured by qRT-qPCR and the nuclear fraction of LXRα evaluated by Western blot. The presented blot derives from the same membrane, the original blot is present in Supplementary Fig. 2. **B** *CLDN5*, *ABCA1*, *OCLDN* mRNA levels measured by qRT-qPCR. **C** CLDN5 localization (white arrows) evaluated via immunostaining. CLDN5 (green) Hoechst (blue). Scale bar 20 µm. **D** *SNAI2* mRNA measured by qRT-PCR and SNAI2 localization (white arrows) evaluated via immunostaining. SNAI2 (red) Hoechst (blue). Scale bar 20 µm. **E** *DLL4* and *NOTCH1* mRNA levels measured by qRT-qPCR. **F** Representative images of sprouting assay of 20% O_2_ (*N* = 23) and 1% O_2_ (*N* = 24) with relative analysis (yellow tracing). Hoechst (blue) and CD31 (green). Scale bar 100 µm. **G** Quantification of total number of sprouts and mean length, maximum and minimum length expressed per condition. The qPCR values were normalized using RPLPO and plotted as fold change of Normoxia. Data presented are the mean of triplicate values of four independent experiments. Statistical analysis was performed using paired Student’s t-test with Welch’s correction *p < 0.05, **p < 0.01, ***p < 0.001.

Reduced tissue oxygen pressure can induce tip cell formation, thereby stimulating sprouting angiogenesis [54]. In light of our previous results reporting enhanced angiogenesis in LXRα KD cells we questioned the role of hypoxia in the regulation of LXRα expression, BECs identity and the angiogenic process. After 48 h in 1% O_2_, BECs maintained their confluence yet showed altered cell morphology (Fig. 5A). Importantly, hypoxia markedly reduced the mRNA (*p* = 0.020) and protein level of LXRα, while expression of the *LXRβ* isoform was largely refractory to this treatment (Fig. 5A). The transcripts of BEC markers *CLDN5* (*p* = 0.034), *ABCA1* (*p* = 0.015) and *OCLDN* (*p* = 0.060) were also downregulated by hypoxia (Fig. 5B). Immunofluorescent detection of CLDN5 in BEC validated the mRNA results by showing that the corresponding protein is also decreased, as its junctional localization (Fig. 5C). A mirror image was observed with *SNAI2*, for which the mRNA expression (*p* = 0.011) and protein levels were increased in hypoxic BECs (Fig. 5D). Interestingly, hypoxic BECs treated with the LXRs pan-agonist GW3965 restored *LXRα* mRNA and protein content to homeostatic levels (Supplementary Fig. 2). Moreover, despite its fundamental role during hypoxia, HIF-1α is not responsible for LXRα suppression as the knockdown of HIF-1α did not rescue LXRα decrease in hypoxic BECs (Supplementary Fig. 2).

To assess the activation of the Notch pathway under hypoxia, we evaluated *DLL4* and *NOTCH1* expression via qPCR and assessed the angiogenic ability using the sprouting assay. Expression of both genes was reduced in hypoxic BECs, however while *DLL4* was significantly downregulated (*p* = 0.033) *NOTCH1* expression did not reach statistical significance (*p* = 0.114) (Fig. 5E). The sprouting assay showed major differences in cells grown in hypoxia (Fig. 5F). These cells had an increased number of sprouts (*p* = 0.001) which were shorter (*p* = 0.001) when compared to control (Fig. 5G). These findings reveal the inhibitory role of hypoxia on LXRα-dependent signalling, which triggers phenotypical changes reminiscent of those observed in LXRα KD cells. Furthermore, the data provide insights on the mechanism of hypoxia-induced angiogenesis.

### SNAI2 is increased in the vasculature of AD patients with capCAA

**Fig. 6:**
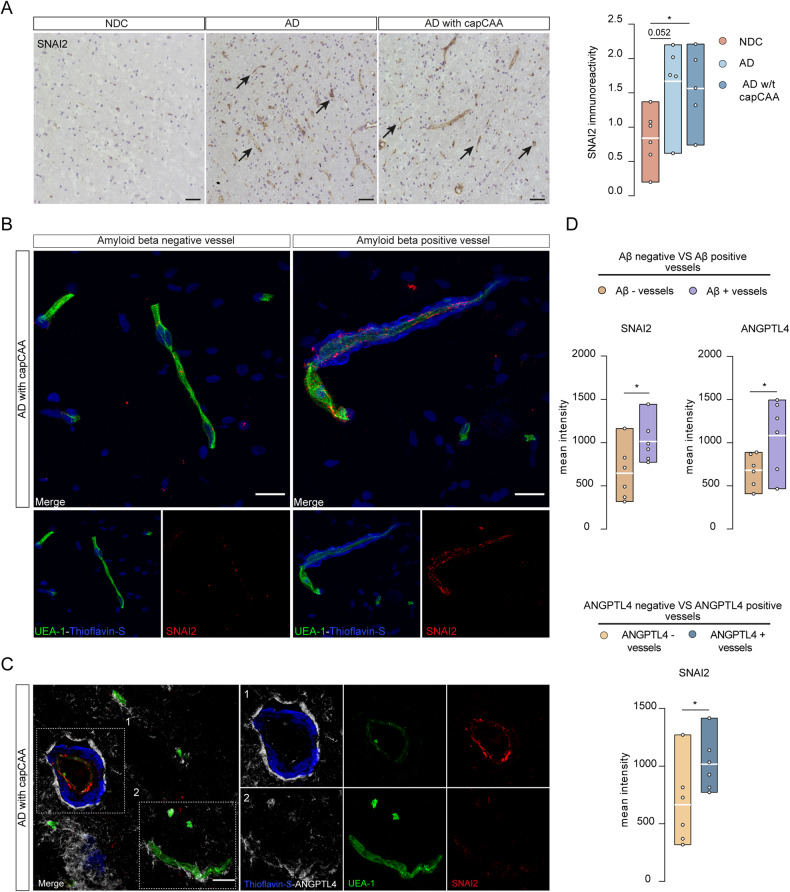
SNAI2 is increased in the vasculature of AD patients with capCAA. **A** SNAI2 reactivity in the cortex of AD (*N* = 5), capCAA (*N* = 5) and NDC (*N* = 6) patients. SNAI2 reactivity in vessels (black arrows). Semiquantitative analysis of SNAI2 reactivity. The values were normalized to the NDC and plotted as fold change. Scale bar 50 µM. **B** Representative image of Aβ positive and negative vessels in AD patients with capCAA (*n* = 6). SNAI2 (red), UEA-1 (green), DAPI and Thioflavin-S (blue). Scale bar 20 µm. **C** Representative images of double positive (Aβ and ANGPTL4) vessels of AD patients with capCAA (*N* = 6). SNAI2 (red), UEA (green), Thioflavin-S (blue), ANGPTL4 (white). Scale bar 20 µm. **D** Mean intensity of ANGPTL4 and SNAI2 in Aβ positive and negative vessels respectively, mean intensity of SNAI2 in ANGPTL4 positive and negative vessels. Statistical analysis was performed using paired Wilcoxon signed-rank test or Mann-Whitney test where *p < 0.05.

As hypoxia and vascular dysfunction strongly associate with AD pathology, we next set out to determine whether SNAl2 participates in the endothelial dysfunction present in AD with and without capCAA [16, 55, 56]. In our immunohistochemical analysis we made use of ANGPTL4 expression in astrocytes as a recently discovered marker of hypoxia in capCAA [57]. Due to the renowned impossibility to stain LXRα in tissue and based on our in vitro data we therefore assessed the localization and expression of SNAI2. We performed immunohistochemistry on the occipital cortex of AD patients with and without capCAA pathology compared to non-demented controls. Immunohistochemical analysis revealed a significant upregulation of SNAI2 in AD patients with capCAA (*p* = 0.038) compared to nondemented controls, while AD patients without capCAA showed only a marginal increase in SNAI2 expression in their vessels (*p* = 0.052) (Fig. 6A). Further immunofluorescent analysis showed that the increased expression of SNAI2 in AD patients with capCAA was associated with vessels affected by Aβ (Fig. 6B).

We next evaluated the implication of hypoxia in the promotion of SNAI2 in AD patients with capCAA, by assessing the expression of ANGPTL4. We confirmed previous results showing that ANGPTL4 is expressed in astrocytes end-feet, primarily in the vicinity of Aβ aggregates (Supplementary Fig. 2). Aβ positive vessels showed a significant increase of ANGPTL4 (*p* = 0.031) compared to vessels negative for Aβ (Fig. 6C and D). The vessels affected by perivascular accumulation of Aβ that were also positive for ANGPTL4 had a significantly higher level of SNAI2 (*p* = 0.031) (Fig. 6C and D). Finally, we evaluated the expression of SNAI2 in ANGPTL4-positive vessels independent of Aβ, and report a consistent increase of SNAI2 (*p* = 0.031) (Fig. 6D). Together, these results suggest that hypoxia induces the expression of SNAI2 in AD patients expressing capCAA pathology.

## Discussion

CapCAA is frequent in AD and is associated with BBB dysfunction, disturbances of cerebral blood flow, and might contribute to cognitive decline [11–16]. However, the underlying mechanism leading to these microvascular changes remain unknown. In our previous study, we showed that LXRα is important in maintaining proper barrier function [41]. In the current study, we decipher how LXRα maintains BEC identity. We report a novel indirect inhibitory activity of LXRα on SNAI2, where the loss of LXRα leads to an increase of SNAI2. This results in the de-differentiation and sprouting of BECs via disruption of the DLL4-Notch axis. Finally, we show that hypoxia affects specifically LXRα expression in vitro and translate our findings to the observed endothelial dysfunction in AD patients with and without capCAA.

Our results suggest that the presence of LXRα is necessary for the constitutive inhibition of SNAI2, where LXRα might compete with LXRβ for the RXRα monomer, which seems the key mechanism for SNAI2 inhibition. *In-silico* ligand-binding kinetic measurements, used to explore the affinity of LXRα/β for RXRα, indicate a stronger interaction for the LXRα-RXRα dimer upon stimulation [51]. These results support our hypothesis of competitive inhibition. However, LXRα has been reported to partner with RXRβ as well [58], which requires us to underline that the proposed mechanism might not be the only active one. Only a few studies so far have addressed the LXR-RXR interaction in such great detail, indicating the necessity to further explore these complex dynamics. LXRs are known to act at the promoter level of specific genes [34, 59, 60]. In hepatocytes, LXRα has been reported to contact SNAI1 promoter thereby regulating its expression [61]. Similarly, LXRβ selectively regulate SNAI3 expression in macrophages, whereas SNAI1 and SNAI2 were not listed among the regulated genes [59]. Recently, the Glass group reported a cell-specific response to LXRs agonist, which may explain the discrepancy between hepatocytes and macrophages on SNAI regulation [62]. Together, these evidence points to an active role for LXRs in SNAI gene regulation. However, further validation is necessary to unambiguously demonstrate that LXRβ-RXRα complex binds the SNAI2 promoter in BECs.

The increase of SNAI2 in LXRα KD cells is accompanied by a loss of endothelial markers. These findings may reflect the initiation of EndMT, which induces dedifferentiation of the BECs [7]. LXRα has been shown to inhibit EMT in myofibroblast [29]. In addition, SNAI2 overexpression was described to induce partial-EndMT in human umbilical vascular endothelial cells [53]. Both SNAI1 and SNAI2 are important transcription factors driving epithelial-to-mesenchymal transition (EMT) [63–65]. However, although SNAI1 also showed a significant increase in LXRα KD cells, the limited fold change increase in LXRα KD cells and the discrepancy with the RNA-seq data, led us to focus on the transcription factor SNAI2.

Moreover, we demonstrate the importance of LXRα in the DLL4-NOTCH axis, further highlighting the role of SNAI2. Where transforming growth factor beta is the main inducer of EndMT via SNAI1, EndMT induction by Notch requires SNAI2 [66, 67]. In addition, the increased expression of SNAI2 is associated with an impaired Notch signalling, marked by DLL4 reduction, KDR (VEGFR2) increase and aberrant angiogenesis [53]. During development the Notch pathway is essential for functional angiogenesis. The leader tip cell expresses DLL4 and signals to the adjacent cells via NOTCH1 to become stalk cells. DLL4-NOTCH1 signalling imposes to the stalk-cells a differential gene expression, ensuring regulated sprouting. In adult vessels, NOTCH1 is necessary to maintain endothelial quiescence [68] and in BECs DLL4-NOTCH signalling regulates their permeability [55–57]. These findings highlight the importance of this pathway for normal vascular behaviour and emphasizes the necessity for a functional LXRα activity.

We report that hypoxia, a known driver of EMT and EndMT [69], inhibits LXRα expression, leaving the β isoform unaffected. This process is not driven by HIF-1α as shown by our in vitro experiments where we combined HIF-1α KD in BECs and hypoxia. Importantly, hypoxic BECs treated with LXRs agonist GW3965 efficiently restored LXRα mRNA and protein content to homeostatic levels. Under hypoxic conditions macrophages drastically reduce cholesterol synthesis leading to the intracellular accumulation of cholesterol esters [70, 71]. In BECs, the diminished cholesterol synthesis might result in reduced oxysterols, which are cholesterol derivatives essential to maintain homeostatic levels of LXRα [72]. The administration of LXRs agonist overcomes the lack of oxysterols and restores LXRα expression. The downregulation of LXRα, but not LXRβ, was also shown in heart tissue of mice after myocardial ischemia [73], which is in line with our findings. Importantly, hypoxic BECs closely recapitulate the changes observed in LXRα KD cells, including increased SNAI2 expression, decreased expression of BEC markers, and impaired DLL4-NOTCH signalling. These results bestow novel molecular insights on the role of hypoxia in BBB structural maintenance, as well as advances our knowledge on angiogenesis.

In the present study we report a vascular increase of SNAI2, associated with partial-EndMT, in vessels of AD patients suffering from capCAA. The general increase of SNAI2 was strongly correlated with the presence of ANGPTL4 and perivascular accumulation of Aβ. The renowned difficulty in visualizing hypoxic genes, including HIF-1α, in post-mortem tissue required an alternative strategy thus we opted for ANGPTL4 as hypoxic marker. ANGPTL4 has been extensively studied in cancer research and reported to increase upon hypoxia and co-localize with HIF-1α [74–76]. In the CNS, ANGPTL4 is expressed by reactive astrocytes in postmortem tissue of patients with capCAA [57]. In line with these results, we report an increase of ANGPTL4 in astrocytes located in the vicinity of Aβ-affected vessel, indicating a local hypoxic environment. Moreover, our results show that Aβ affected vessels marked by ANGPTL4 have increased SNAI2 expression, possibly leading to partial-EndMT and angiogenesis. Whether Aβ is the driving force inducing localized hypoxia is still unclear and much debated [77]. It is important to mention that the lack of immunohistochemical validation of LXRα expression in AD patients with capCAA is a limitation of this study. However, the technical challenges posed by the lack of reliable IHC antibodies forced us to abandon this validation and focus on SNAI2.

The formation of new vessels is a delicate process with many facets. During stroke for instance, angiogenesis takes place rapidly after injury [78, 79] and associates with higher survival rate [80]. However, in the diabetic retina aberrant angiogenesis results in haemorrhage and oedema [81], emphasizing the double role exerted by this mechanism. In AD, cerebrovascular defects including reduced cerebral blood flow [16, 55, 56] correlate with higher vessel number [56, 57, 82, 83] suggesting reduction in brain oxygen concentration and angiogenesis promotion. This process is accentuated in capCAA vessels, where Aβ accumulation further exacerbates hypoxia, leading to partial-EndMT and angiogenesis. Nevertheless, whether this is a detrimental mechanism or a reparative program activated to re-establish the brain’s oxygen supply is still unclear and requires further study. Several studies have reported a beneficial effect of LXR agonists treatment in AD mouse models. LXRs activation favors Aβ clearance, supports microglial Aβ phagocytosis and promotes cognitive recovery [84–86]. In addition, short-term administration of GW3965 exerts a beneficial effect on the brain vasculature of aged 3xTg-AD mice [87]. Importantly, a selective LXR agonist or antagonist is at this time unavailable, making the systemic side effects due to LXRα activation (e.g. liver steatosis) the major challenge for clinical translation.

To conclude, we uncovered a new regulatory mechanism of LXRα, which is essential to preserve BEC identity. We propose that in AD the increased vascular resistance and reduced cerebral blood flow result in chronic brain hypoxia. This results in increased SNAI2 expression and concomitant brain endothelial remodelling, although more in-depth experiments are necessary to confirm this. Understanding the molecular mechanism underlying BBB impairment in AD is crucial to better define the pathological progression and develop disease-modifying therapies.

### Supplementary information

Figure and Table legends

Supplementary figure 1

Supplementary figure 2

Supplementary Table. 1

Supplementary Table. 2

Supplementary Table. 3

Supplementary Table. 4

Western blot LXRA in LXRA KD cells

Western blot LXRa under hypoxia

Reproducibility check list

## Data Availability

All data generated or analysed during this study are included in this published article Supplementary Tables 2 and 3.
