# Supplementary material for: Liver X receptor alpha ensures blood-brain barrier function by suppressing SNAI2
Source: Cell Death Dis. 2023 Nov 28;14(11):781. doi: 10.1038/s41419-023-06316-8 (PMC10684660; doi:10.1038/s41419-023-06316-8)
Supplement: Supplementary file 1 — Figure and Table legends [file 41419_2023_6316_MOESM1_ESM.docx]

**Supplementary figure 1.**

A) Heat-map of KEGG pathways of cell adhesion molecules and ABC transporter family of NTC vs LXRα KD hCMEC/D3 cells. B) Knock-down efficiency on protein level in brain endothelial cells transduced with LXR alpha lentivirus. Nuclear protein content of LXRα in non-targeting control (NTC) and LXRα KD BEC was assessed by western blot, indicating a decreased expression of LXRα in LXRα KD cells compared to NTC (N=1) C) The mRNA levels of JAG1, JAG2 measured by qRT-qPCR in LXRα KD cells. D) The mRNA levels of LXRα, DLL4 and NOTCH1 measured by qRT-qPCR in LXRα/β KD cells treated with GW3965 (1µM for 48h). The qPCR values were normalized using GAPDH and plotted as fold change of control (NTC). Data presented are the mean of triplicate values of three independent experiments. Statistical analysis was performed using paired Student’s t-test with Welch’s correction where * p< 0.05, ** p<0.001.

**Supplementary figure 2**.

A) The mRNA levels *LXRα* , *ABCA1*, *LXRβ* of measured by qRT-qPCR in hypoxic hCMEC/D3 cells (1% O_2_  ± GW3965 for 48h). B) Original western blot of LXRα nuclear fraction of hypoxic hCMEC/D3 cells treated with GW3965 (1% O_2_  ± GW3965 for 48h). C) The mRNA levels of *JAG1*, *JAG2* and *HES1* measured by qRT-qPCR in hypoxic hCMEC/D3 cells (1% O_2_ for 48h). D) mRNA levels of *HIF-1α*, *LXRα* and *SNAI2* measured by qRT-qPCR in HIF-1α KD cells (20% O_2_ or 1% O_2_ for 48h). The qPCR values were normalized using RPLPO and plotted as fold change of normoxia or NTC. Data presented are the mean of triplicate values of three or four independent experiments. E) Representative image of sprouting endothelial cells expressing CD31 and Zonulin-1. DAPI (Blue), CD31 (Green) Zonulin-1 (ZO-1) (Red). F) Representative image of Aβ affected vessels surrounded by reactive astrocytes expressing ANGPTL4. UEA-1 (Green), Thioflavin-S (Blue), ANGPTL4 (Cyan) and GFAP (Red). Statistical analysis was performed using paired Student’s t-test with Welch’s correction where * p< 0.05.

**Supplementary Table 1.** Primer sequence.

**Supplementary Table 2.** Gene set enrichment analysis of LXRα KD cells and NTC.

**Supplementary Table 3.** RNA-seq results of LXRα KD cells and NTC.

**Supplementary Table 4.** Patients data. Non-demented control (NDC), Alzheimer’s disease (AD), Alzheimer’s disease with capCAA (AD with capCAA), post-mortem delay (PMD).
