# Supplementary figures and images for: Liver X receptor alpha ensures blood-brain barrier function by suppressing SNAI2
Source: Cell Death Dis. 2023 Nov 28;14(11):781. doi: 10.1038/s41419-023-06316-8 (PMC10684660; doi:10.1038/s41419-023-06316-8)

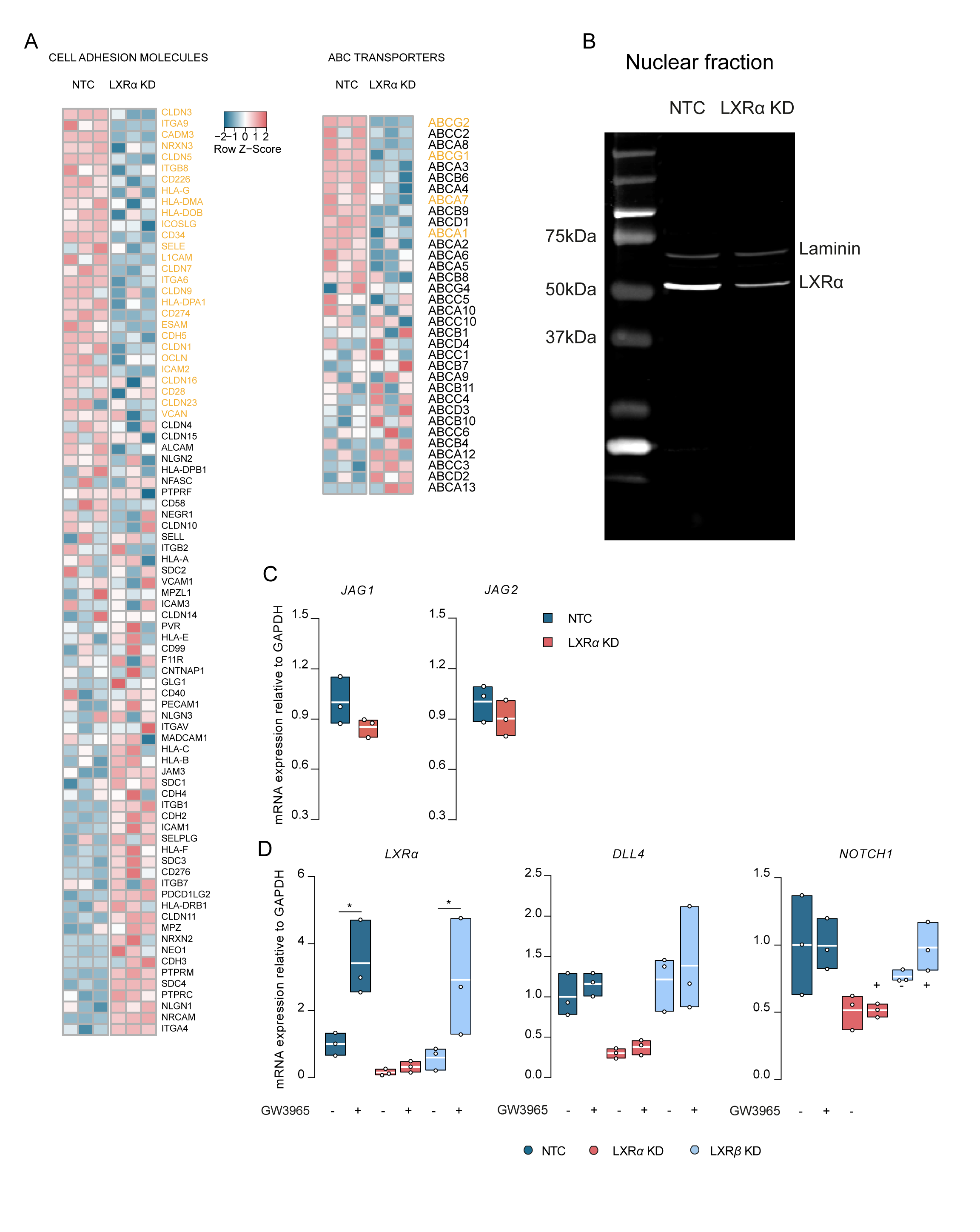

Supplement: Supplementary file 2 — Supplementary figure 1 [file 41419_2023_6316_MOESM2_ESM.tif]

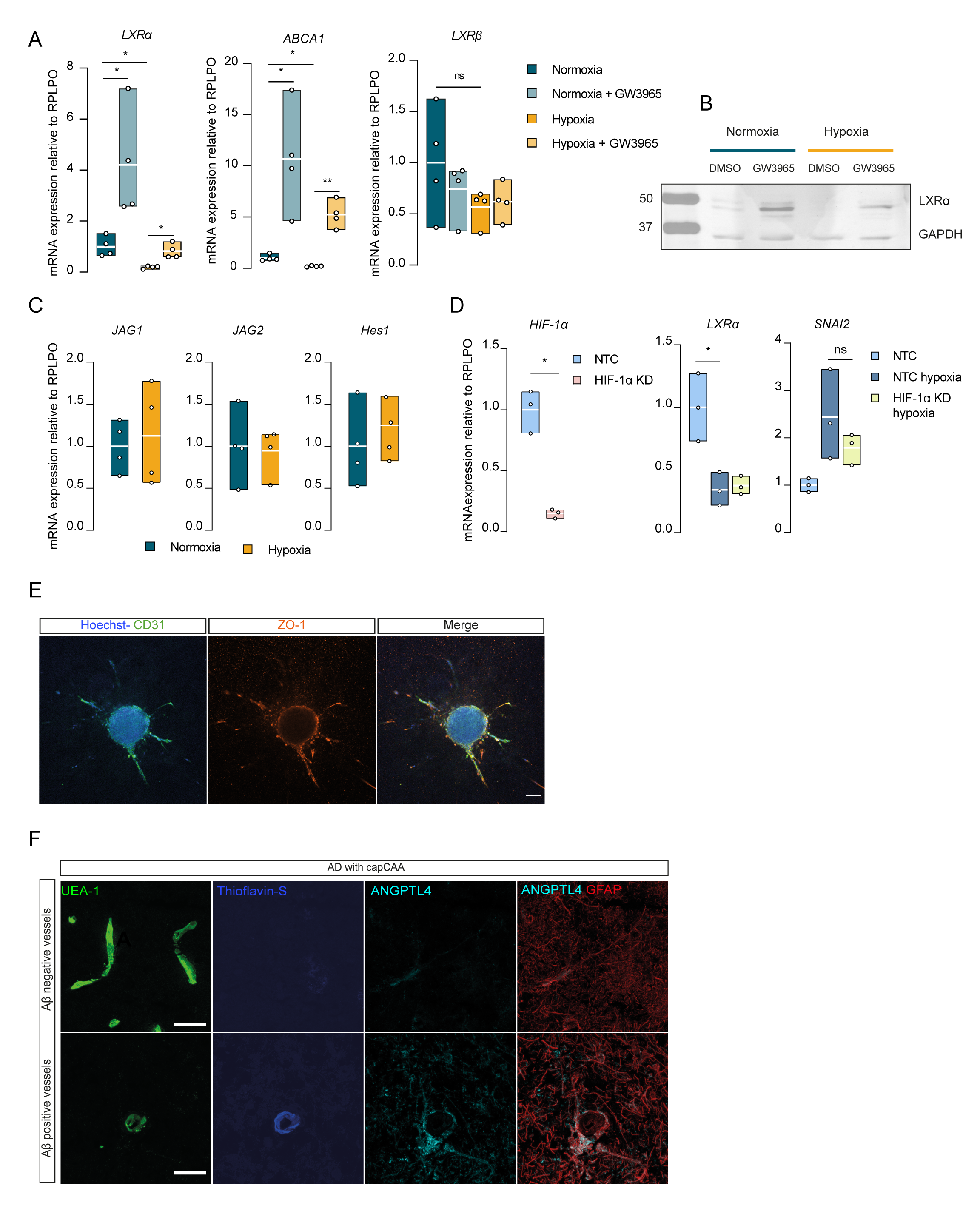

Supplement: Supplementary file 3 — Supplementary figure 2 [file 41419_2023_6316_MOESM3_ESM.tif]

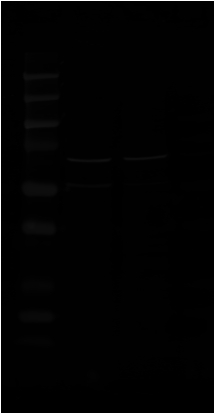

Supplement: Supplementary file 8 — Western blot LXRA in LXRA KD cells [file 41419_2023_6316_MOESM8_ESM.tif]

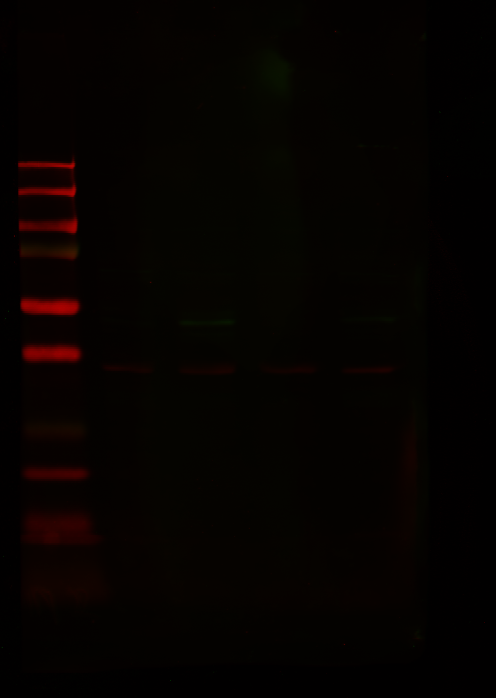

Supplement: Supplementary file 9 — Western blot LXRa under hypoxia [file 41419_2023_6316_MOESM9_ESM.tif]
